# Alemtuzumab in the long-term treatment of relapsing-remitting multiple sclerosis: an update on the clinical trial evidence and data from the real world

**DOI:** 10.1177/1756285617722706

**Published:** 2017-08-04

**Authors:** Tjalf Ziemssen, Katja Thomas

**Affiliations:** Center of Clinical Neuroscience, Carl Gustav Carus University Clinic, Dresden University of Technology, Dresden, Germany; Center of Clinical Neuroscience, Carl Gustav Carus University Clinic, Dresden University of Technology, Dresden, Germany

**Keywords:** alemtuzumab, mechanism of action, multiple sclerosis, long-term efficacy, long-term safety, real-world experience

## Abstract

Alemtuzumab is a humanized monoclonal antibody approved for the treatment of relapsing-remitting multiple sclerosis (RRMS), given as two annual courses on five consecutive days at baseline and on three consecutive days 12 months later. Here we provide an update on the long-term efficacy and safety of alemtuzumab in RRMS, including real-world experience, and advances in our understanding of its mechanism of action. Recent data from the phase II/III extension study have demonstrated that alemtuzumab reduces relapse rates, disability worsening, and the rate of brain volume loss over the long term, with many patients achieving no evidence of disease activity. In high proportions of patients, preexisting disability remained stable or improved. Alemtuzumab is associated with a consistent safety profile over the long term, with no new safety signals emerging and the overall annual incidence of reported adverse events decreasing after the first year on treatment. Acyclovir prophylaxis reduces herpetic infections, and monitoring has been shown to mitigate the risk of autoimmune adverse events, allowing early detection and overall effective management. Data from clinical practice and ongoing observational studies are providing additional information on the real-world use of alemtuzumab. Recent evidence on the mechanism of action of alemtuzumab indicates that in addition to its previously known effects of inducing depletion and repopulation of T and B lymphocytes, it also results in a relative increase of cells with memory and regulatory phenotypes and a decrease in cells with a proinflammatory signature, and may further promote an immunoregulatory environment through an impact on other innate immune cells (e.g. dendritic cells) that play a role in MS. These effects may allow preservation of innate immunity and immunosurveillance. Together, these lines of evidence help explain the durable clinical efficacy of alemtuzumab, in the absence of continuous treatment, in patients with RRMS.

## Introduction

Alemtuzumab is a humanized monoclonal antibody licensed in over 60 countries for the treatment of patients with relapsing-remitting multiple sclerosis (RRMS). The efficacy and safety of two annual courses of alemtuzumab 12 mg were evaluated in three randomized, head-to-head, rater-blinded, clinical trials against subcutaneous (SC) interferon (IFN)β-1a 44 μg three times per week in patients with active RRMS. Overall, two of the trials included patients who were treatment-naïve (phase II CAMMS223 study [ClinicalTrials.gov identifier: NCT00050778] and phase III Comparison of Alemtuzumab and Rebif^®^ Efficacy in Multiple Sclerosis [CARE-MS I; ClinicalTrials.gov identifier: NCT00530348]), whereas the third study included patients who had an inadequate response to prior therapy (phase III CARE-MS II [ClinicalTrials.gov identifier: NCT00548405]).^1–3^ Alemtuzumab significantly reduced relapses by 49−74% compared with SC IFNβ-1a (*p* < 0.001 in all studies), and significantly reduced confirmed disability worsening (CDW) in the CAMMS223 (hazard ratio, 0.29; *p* < 0.001) and CARE-MS II trials (hazard ratio, 0.58; *p* = 0.008). Patients in CARE-MS I demonstrated a nonsignificant 30% reduction in risk of CDW (hazard ratio, 0.70; *p* = 0.22). In addition to these endpoints, alemtuzumab improved preexisting disability and reduced the rate of brain volume loss (BVL), with many patients achieving no evidence of disease activity (NEDA) on an annual basis. The long-term efficacy and safety of alemtuzumab were investigated in an extension of these clinical trials [ClinicalTrials.gov identifier: NCT00930553], demonstrating durable efficacy with no new safety signals.^4,5^ The most common adverse events (AEs) in alemtuzumab clinical studies were infusion-associated reactions (IARs).^1–3^ Autoimmune AEs, most commonly affecting the thyroid, but with infrequent cases of immune thrombocytopenia (ITP), nephropathies, and infections, were also reported. Autoimmune events can generally be managed with active monitoring for early detection and prompt clinical attention.^6,7^

The dosing schedule for alemtuzumab, two courses comprising intravenous infusion of 12 mg/day on five consecutive days at baseline and three consecutive days 12 months later (96 mg total dose), is distinct from other MS therapies.^6,8–10^ In the clinical trials, additional treatment courses of 12 mg/day on three consecutive days could be administered at least 12 months after the prior course upon documented evidence of MS disease activity and per discretion of the treating physician; however, many patients did not receive any additional treatment after the initial two courses of alemtuzumab. In contrast with other disease-modifying therapies (DMTs) approved for RRMS, which require dosing either daily, every other day, thrice weekly, weekly, every other week, monthly, or every 6 months, alemtuzumab is the only approved treatment that does not require regular dosing in RRMS patients.

With newly available data from the CARE-MS extension study, and several years of post-approval, real-world use, our understanding of alemtuzumab’s long-term effects is increasing. This article reviews the evidence for long-term efficacy and safety of alemtuzumab, presents the data from real-world experience, and discusses advances in our understanding of the mechanism of action of alemtuzumab in RRMS. Up to know, 13.000 MS patients have been treated with alemtuzumab.

## Efficacy in long-term clinical trials

### Study enrollment, retention, and alemtuzumab retreatment rates

Patients who completed the core phase II and III studies were eligible to enroll in a long-term extension study in which they could receive as-needed retreatment with alemtuzumab, as determined by the treating physician in consultation with the patient. In order to receive alemtuzumab retreatment, patients were required to meet specific disease activity criteria: ⩾1 protocol-defined relapse, or ⩾2 new/enlarging T2 hyperintense or new gadolinium (Gd)-enhancing T1 brain or spinal cord lesions on magnetic resonance imaging (MRI), and have none of the prespecified disqualifications for retreatment (mainly pregnancy, malignancy, certain infections, and autoimmune conditions). Other DMTs could be administered at the investigator’s discretion.^11^ Alemtuzumab retreatment was unavailable for part of the CAMMS223 study follow up during a dosing suspension (2005−2008).

For the CARE-MS studies, data are available for 6 years of treatment (2 years of the core study plus 4 years of the extension). In CARE-MS I, 376 patients were treated with alemtuzumab in the core study, 349 entered the extension, and 325 remained on study through Year 6, for a retention rate of 86% from core study baseline and 93% from enrollment in the extension. In CARE-MS II, 435 patients were treated with alemtuzumab in the core study, 393 entered the extension, and 344 remained on study through Year 6, with retention rates of 79% from core study baseline and 88% from extension study baseline.^4,5^

The CARE-MS study retention rates are higher than those reported for other DMTs after long-term follow up over 4–5 years. For patients on fingolimod in the TRANSFORMS extension study, retention was 62% (526/849) from the core study baseline and 77% (526/687) from the extension study baseline^12^; the FREEDOMS extension study showed 63% (535/854) and 86% (535/620) retention, respectively.^13^ In ENDORSE, for patients on dimethyl fumarate, retention rates were 47% (720/1530) from core study baseline and 72% (720/1003) from extension study baseline.^14–16^ Loss to follow up in randomized controlled trials may bias results if unavailable data are associated with the likelihood of outcome events, and lead to both over- and under-estimation of treatment effects.^17^ The high retention rate of the CARE-MS extension study suggests that the long-term efficacy data for alemtuzumab are unusually robust compared with data from trials of other DMTs with higher dropout rates.

For the CAMMS223 study, patients completing the 3-year core study could enter an extended follow-up period (minimum additional 2 years), and then in 2010 they could enroll in the same extension study as patients from the CARE-MS studies, for a total available follow up of 10 years. Of the 108 patients who received alemtuzumab 12 mg in CAMMS223, 92 completed the core study, 72 entered the extended follow-up period, and 60 patients entered the CARE-MS extension; 57 remained on study at Year 10.^18^

**Figure 1.**
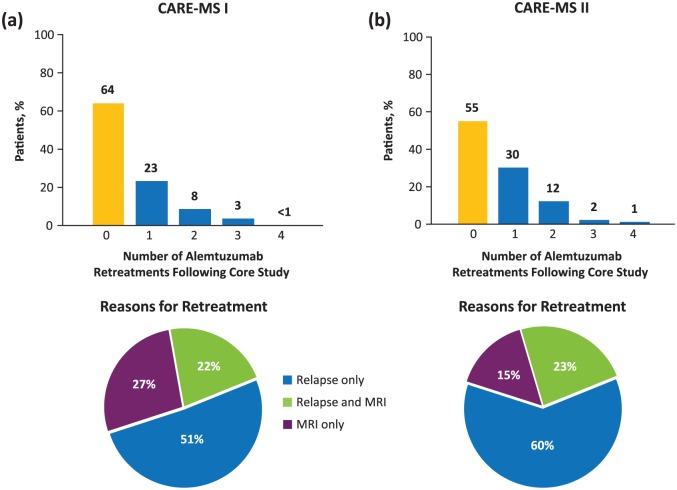
Through Year 6 of the CARE-MS extension, in cohorts of patients entering from (a) CARE-MS I and (b) CARE-MS II, the majority of patients received no further alemtuzumab after their second course.^4,5^ CARE-MS, Comparison of Alemtuzumab and Rebif^®^ Efficacy in Multiple Sclerosis; DMT, disease-modifying therapy; MRI, magnetic resonance imaging.

Many patients from the randomized controlled trials did not receive additional alemtuzumab during the extension (Figure 1). In CARE-MS I, 64% of patients required only the initial two courses of alemtuzumab, with 23%, 8%, 3%, and <1% receiving a third, fourth, fifth, or sixth course, respectively, over 6 years.^4^ Similarly, in CARE-MS II, 55% of patients required only the initial two courses of alemtuzumab with 30%, 12%, 2%, and 1% receiving a third, fourth, fifth, or sixth course, respectively.^5^ Of the patients from CAMMS223 who entered the CARE-MS extension, 33% received only the initial two courses of alemtuzumab over 10 years; 43%, 12%, and 10% received three, four, or five courses of alemtuzumab, respectively.^18^ Across the three studies, investigators cited relapse as the most common reason for retreatment.

### Annualized relapse rate (ARR)

Relapse reduction with alemtuzumab was durable over the long term in the CARE-MS extension (Table 1). For patients from CARE-MS I, ARR was 0.16 during the randomized, controlled portion of the trial (Years 0−2) and 0.15 at the end of the extension portion (Years 3−6); for patients from CARE-MS II, ARR was 0.28 and 0.20 in these time intervals, respectively.^4,5^ Among patients originating from the CAMMS223 study, an ARR of 0.07 in the core study (Year 3) was maintained through Year 10 (0.08).^18^

**Table 1.** Key outcomes at completion of core study (Year 2) and CARE-MS Extension (Year 6).

Endpoint	CARE-MS I		CARE-MS II	
	Year 2	Year 6	Year 2	Year 6
ARR^a^	0.16	0.15	0.28	0.20
CDW-free (%)	92	77	89	72
EDSS improved/stable (%)	89	81	85	77
NEDA (annual) (%)	68	57	58	60
Clinical disease activity-free (%)	85	86	73	85
MRI disease activity-free (%)	77	66	76	69
New T2 lesion-free (%)	78	67	76	69
New Gd-enhancing lesion-free (%)	93	87	91	91

a Cumulative ARR for Years 0−2 and Years 3−6.^4,5,22,23,27^

ARR, annualized relapse rate; CARE-MS, Comparison of Alemtuzumab and Rebif^®^ Efficacy in Multiple Sclerosis; CDW, confirmed disability worsening; EDSS, Expanded Disability Status Scale; Gd, gadolinium; MRI, magnetic resonance imaging; NEDA, no evidence of disease activity.

### Disability

**Figure 2.**
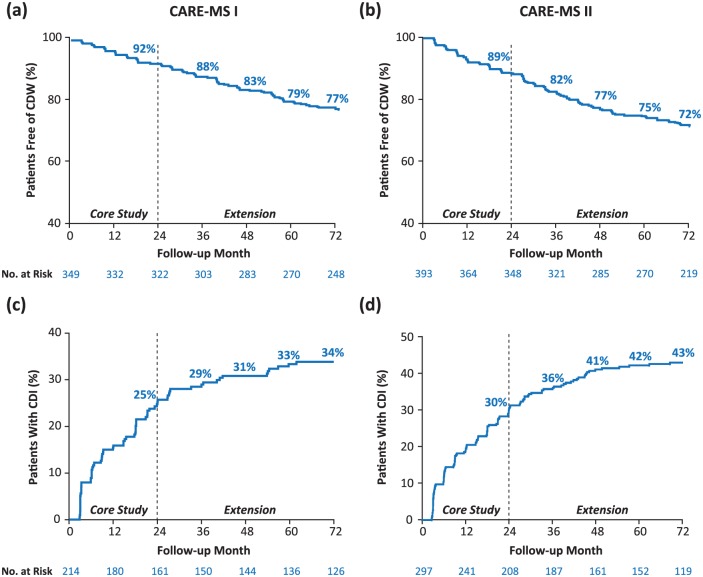
(a, b) The 6-month CDW and (c, d) 6-month CDI through 6 years in patients from CARE-MS I and II.^4,5^ Number at risk is the number of patients who remained on study and who had yet to experience CDW. For CDI analyses, number at risk is the number of patients with baseline EDSS of ⩾2.0, who remained on study, and who had yet to experience CDI. CARE-MS, Comparison of Alemtuzumab and Rebif^®^ Efficacy in Multiple Sclerosis; CDI, confirmed disability improvement; CDW, confirmed disability worsening; EDSS, Expanded Disability Status Score

At Year 2, 85−89% of patients in the CARE-MS studies had stable [⩽0.5-point Expanded Disability Status Scale (EDSS) change from baseline] or improved (⩾1.0-point decrease from baseline) disability; whereas at Year 6, 77−81% of patients had stable or improved disability.^4,5,19^ Through Year 6, 72−77% of patients were free of 6-month CDW [defined as an increase of ⩾1.0 EDSS point (or ⩾1.5 points if baseline EDSS = 0)], and 34−43% of patients achieved 6-month confirmed disability improvement (CDI; defined as ⩾1-point EDSS decrease from baseline, assessed in patients with baseline score ⩾2.0; Figure 2).^4,5^ The proportion of patients with CDI increased from Year 2 to Year 6, indicating that patients continued to experience CDI events during the extension study. Furthermore, of the patients who achieved 6-month CDI, 91−94% were free from 6-month CDW, indicating that the improvement in disability was not followed by worsening.

Patients from CAMMS223 demonstrated that alemtuzumab stabilized disability over a 10-year timeframe. The mean EDSS score change from baseline to Year 10 (standard deviation) was +0.12 (1.4); disability scores remained stable or improved in 78% of alemtuzumab-treated patients, and most patients (76%) remained free of 6-month CDW through 10 years.^18^

An analysis of CARE-MS I and II estimated the proportion of patients who converted from RRMS to secondary progressive MS (SPMS) following alemtuzumab treatment, using a definition of SPMS recently developed by Lorscheider and colleagues based on data from the MSBase registry.^20,21^ Low rates of conversion were observed over 6 years in CARE-MS I (1.1%) and CARE-MS II (3.7%).^21^ Notably, the rate of SPMS conversion among the MSBase cohort was 18% over 5.8 years’ follow up.^20^

### MRI lesions

In the CARE-MS studies, freedom from MRI disease activity was defined as an absence of new Gd-enhancing lesions and new/enlarging T2 hyperintense lesions. In CARE-MS I, 77% of patients were free of MRI disease activity in Year 2, and this effect was durable in Years 3−6, during which 66−72% of patients were free of MRI disease activity in each year. MRI disease activity was driven by T2 hyperintense lesions, as the proportion of patients without new Gd-enhancing lesions on their annual scan consistently remained near 90% in each year (range over Years 3−6: 87−90%, compared with 93% in Year 2). Furthermore, most patients had no new nonenhancing T1 hypointense lesions (range over Years 2−6: 82−93%).^22^ Results were similar in CARE-MS II: 72% of patients were free of MRI disease activity in Year 2, and this proportion remained essentially unchanged in Years 3−6 (68−70%); 91% were free of Gd-enhancing lesions on their Year 2 scan, remaining similarly high in Years 3−6 (87−91%). Annually in Years 2−6, 86−93% had no new nonenhancing T1 hypointense lesions.^23^

Data from the 10-year cohort of CAMMS223 confirmed that alemtuzumab is highly effective at reducing Gd-enhancing lesion activity. At CAMMS223 baseline, Gd-enhancing lesions were present in 100% of patients, reflecting the study inclusion criteria. After alemtuzumab treatment, only 8.3% of patients had Gd-enhancing lesions upon entry into the CARE-MS extension (roughly Year 5 of follow up), with 5.6%, 13.0%, and 5.5% of patients having Gd-enhancing lesions in the first, second, and third years of the CARE-MS extension study, respectively.^24^ Other MRI outcomes are not yet reported for this cohort.

### No evidence of disease activity (NEDA)

NEDA (freedom from relapses, 6-month CDW, new Gd-enhancing lesions, and new/enlarging T2 hyperintense lesions) is a composite endpoint that has come into use in recent years as a high standard for DMT efficacy.^25^ The proportion of alemtuzumab-treated patients achieving NEDA was consistent in each year of the extension through 6 years. Of patients from CARE-MS I, most achieved annual NEDA: Year 2, 68%; Year 3, 62%; Year 4, 60%; Year 5, 62%; Year 6, 57%.^26^ Results were similar for CARE-MS II (Year 2, 58%; Year 3, 53%; Year 4, 55%; Year 5, 58%; Year 6, 60%).^27^

### Brain volume loss (BVL)

**Figure 3.**
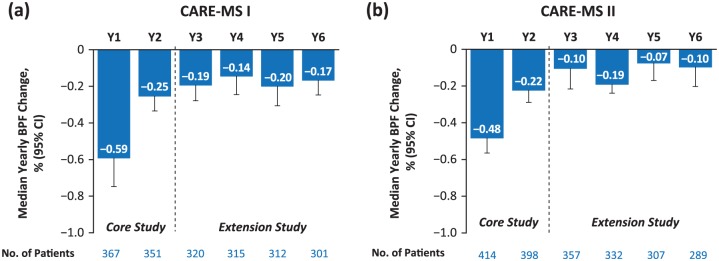
Median annual change in brain volume, measured by BPF, in alemtuzumab-treated patients enrolled in the extension from CARE-MS I (a) or II (b).^32^ BPF, brain parenchymal fraction; CARE-MS, Comparison of Alemtuzumab and Rebif^®^ Efficacy in Multiple Sclerosis; Y, year.

Brain atrophy, as measured by BVL, is a concomitant of physiologic aging. The average rate of annual BVL in healthy adults is age-dependent and heterogeneous among different tissue compartments; annual brain parenchymal loss ranges from −0.20% at age 35 to −0.52% at age 75.^28^ Pathologic BVL occurs very early in the MS disease course and is predictive of disability and cognitive impairment.^29–31^ In the alemtuzumab core and extension studies, BVL was derived from the change from baseline in brain parenchymal fraction, calculated from proton density/T2-weighted dual-echo images. Median yearly BVL progressively decreased over 2 years in alemtuzumab-treated patients from CARE-MS I and remained low in Years 3−6 (range, −0.14 to −0.20%); similar results were observed in CARE-MS II (median yearly BVL in Years 3−6 range, −0.07 to −0.19%; Figure 3).^32^

### Quality of life (QoL)

QoL assessments are an important complement to clinical and MRI data as they provide a measure of therapeutic effectiveness from the patient’s perspective.^33^ Overall, three validated instruments were used to assess QoL during alemtuzumab studies: the Functional Assessment of Multiple Sclerosis (FAMS), a questionnaire specific to MS that assesses mobility, symptoms, emotional wellbeing, general contentment, thinking, fatigue, and family/social wellbeing; the 36-Item Short-Form Survey (SF-36), which is summarized into mental and physical component summary (MCS resp. PCS) scores; and the European QoL 5 Dimension (EQ-5D) visual analog scale, which is a single-question rating from 0–100. In all three questionnaires, a higher score indicates better functioning.^34–36^

Alemtuzumab significantly improved QoL over 2 years during both core CARE-MS studies compared with baseline and with SC IFNβ-1a. Gains in QoL measures paralleled clinical and radiologic benefits. Analyses over 6 years of follow up have been reported for CARE-MS II. At the end of the randomized, controlled CARE-MS II trial (Year 2), alemtuzumab patients had significant improvements from baseline on FAMS [change from baseline at Year 2, 6.20 (95% confidence interval (CI): 3.69–8.71)], SF-36 MCS [2.07 (95% CI: 1.03–3.11)], SF-36 PCS [2.51 (95% CI: 1.66–3.36)], and EQ-5D visual analog score [4.29 (95% CI: 2.51–6.08)].^37^ At Year 6, CARE-MS II alemtuzumab patients maintained statistically significant improvements from baseline on FAMS [change from core study baseline, 3.16 (95% CI: 0.07–6.25)], SF-36 MCS [1.58 (95% CI: 0.35–2.82)], and EQ-5D [3.16 (95% CI: 0.93–5.38)], and nonsignificant improvements on SF-36 PCS [1.0 (95% CI: –0.03–2.03)].^38^

**Figure 4.**
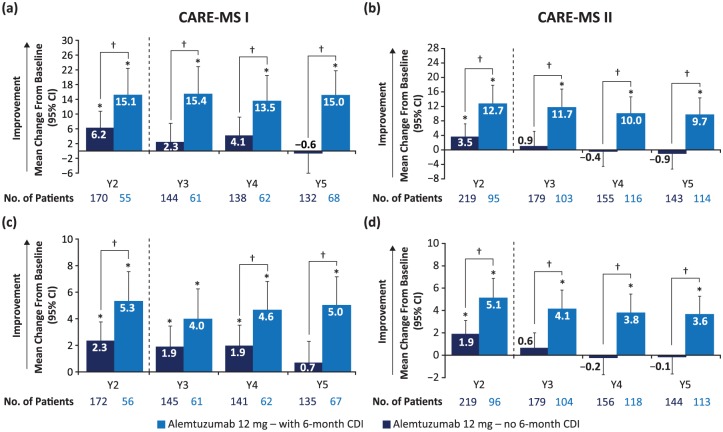
Change from baseline in FAMS score (a, b) and SF-36 PCS (c,d) score in CARE-MS I and II patients with and without 6-month CDI.^39,45^ *Significant change from baseline, *p* < 0.05; †Significant difference between groups, *p* < 0.05. CARE-MS, Comparison of Alemtuzumab and Rebif^®^ Efficacy in Multiple Sclerosis; CDI, confirmed disability improvement; FAMS, Functional Assessment of Multiple Sclerosis; PCS, Physical Component Summary; SF-36, 36-Item Short-Form Survey; Y, year.

The clinical meaningfulness of improvements in QoL and their link to disability outcomes was demonstrated in a 5-year analysis in patients who did or did not experience 6-month CDI. Mean QoL improvements from baseline were significantly greater in patients who achieved CDI *versus* those who did not in Years 2, 3, 4, and 5 for FAMS, EQ-5D visual analog scale, and SF-36 PCS, and in Year 5 for SF-36 MCS (Figure 4).^39^

## Safety in long-term clinical trials and in real-world experience

### Infusion-associated reactions (IARs)

The most common AEs with alemtuzumab are IARs, which are mostly mild-to-moderate, and most commonly consist of rash, headache, pyrexia, urticaria, and nausea.^40,41^ In the pooled CARE-MS studies (*n* = 811), the incidence of IARs decreased with successive courses of alemtuzumab (courses 1−5: 85%, 69%, 65%, 63%, and 46%, respectively). IARs are manageable with appropriate pretreatment using methylprednisolone, symptomatic treatment during and after infusion, and infusion rate adjustment. IARs are not increased by modest changes in the infusion routine (i.e. administration on nonconsecutive days).^42^ In the clinical trials, a serious AE was defined as fatal or life-threatening; requiring or prolonging hospitalization; causing persistent or significant disability/incapacity or congenital anomaly; or requiring intervention to prevent permanent impairment or damage. Serious IARs occurred in ⩽2% of patients during each course.^40^

### Infections

Although infections during the CARE-MS core studies were observed more frequently with alemtuzumab than with the active comparator, SC IFNβ-1a,^1,3^ the incidence of infection was highest after the first treatment course and subsequently declined. In CARE-MS I, incidence was 56.1% in Year 1, declining to 35.5% in Year 6. In CARE-MS II, infection incidence was 63.2% in Year 1 and declined to 43.4% in Year 6. Rates of serious infections were low in each year (<2% in CARE-MS I and <3% in CARE-MS II).^4,5^

After a protocol amendment in the core studies, acyclovir was given during the first month after each course of alemtuzumab as prophylaxis for herpetic infections. Herpetic infection data are available for the pooled CARE-MS core studies and the first 2 years of the extension. In Years 1−4, herpes simplex incidence was 9.0%, 5.4%, 3.1%, and 2.5%, respectively, and herpes zoster incidence was 2.1%, 2.7%, 3.4%, and 2.2%, respectively.^43^

Outside the clinical trials, several case studies have reported opportunistic infections after alemtuzumab treatment. Overall, two patients developed cytomegalovirus disease ⩽1 month after the initial 5-day course and recovered fully with treatment.^44^ Among 66 patients who received a total of 100 courses of alemtuzumab during 1 year at a tertiary center in Germany, 3 developed acute acalculous cholecystitis.^45^ All three patients recovered rapidly following treatment. In the United States (US), a rare pathogen, *Nocardia beijingensis*, was isolated from a patient diagnosed with sepsis secondary to pneumonia 6 weeks after completing the first course of alemtuzumab; the patient responded well to treatment.^46^ Finally, several cases of *Listeria monocytogenes* infection have been reported. In one case, the patient had recent exposure to foods including smoked sausage and soft cheese and developed *Listeria* septicemia after the initial alemtuzumab infusion.^47^ Additionally, three cases of *Listeria* meningitis occurred shortly after the first course of alemtuzumab and were successfully managed, although one patient had severe sequelae.^48,49^

Postmarketing surveillance data on opportunistic infections, gathered by the manufacturer on the ~13,000 patients worldwide who have been treated with alemtuzumab for MS, have been reported.^50^ The most commonly reported opportunistic infection is *L. monocytogenes* (estimated frequency: 0.26%), followed by cytomegalovirus (estimated frequency: 0.13%). Product labeling now recommends that patients avoid or adequately heat foods that are potential sources of *Listeria* in a period of 2 weeks before and 2 months after alemtuzumab infusion. There have been no medically confirmed cases of progressive multifocal leukoencephalopathy (PML) in MS without relevant pretreatments (natalizumab-associated PML).

### Autoimmune events

Alemtuzumab has been associated with autoimmune AEs due, it is hypothesized, to its mechanism of action. These AEs, mainly comprising thyroid disorders and, less frequently, ITP and nephropathies, can occur early post-treatment; however, onset is more often delayed a few years after the last infusion.^51^ The clinical development program for alemtuzumab included a number of steps to mitigate risks associated with these AEs; protocol-defined laboratory monitoring measures and physician and patient education facilitated early detection and effective management of the conditions. These measures have also been implemented in clinical practice as part of the Risk Evaluation and Mitigation Strategy (US) and Risk Management Plan (Europe), providing for monitoring beginning prior to treatment and continuing for 48 months after the last dose of alemtuzumab, or longer if clinically indicated.^8,10^

Among pooled CARE-MS patients who received alemtuzumab 12 mg (*n* = 811), the cumulative incidence of thyroid events was 42% over 6 years.^52^ First onset of the majority (82%) of thyroid AEs occurred within 2 years of the last alemtuzumab treatment course. For events related to hyperthyroidism, treatments included anti-thyroid medications, radioiodine ablation, or thyroidectomy (combined with thyroid hormone replacement therapy); hypothyroid conditions were treated with thyroid hormone replacement therapy. In patients with thyroid AEs, 20.7% of cases resolved spontaneously (i.e. without treatment); of those who were treated according to site protocols, 79.0% (*n* = 271) received conventional oral medications, 9.0% (*n* = 31) had iodine ablation, and 8.5% (*n* = 29) underwent thyroidectomy (some received more than one treatment). Few serious thyroid AEs were reported; the incidence of serious thyroid AEs peaked at Year 3 (3.1%) and subsequently declined.

Data on ITP in the clinical development program are available with a December 2014 cutoff date, representing roughly 5 years of total follow up for the CARE-MS studies and 8 years for CAMMS223.^53^ The cumulative incidence of ITP was 2.3% (*n* = 34 of 1486) in all alemtuzumab-treated patients, and 2.0% (*n* = 24 of 1217) in those treated with the 12-mg dose. Across the clinical development program, most of the 34 patients who developed ITP received only first-line therapy, consisting of corticosteroids with or without intravenous immunoglobulin or platelet infusion. A total of seven patients required second-line therapy with rituximab, and one patient was treated with splenectomy according to the site treatment protocol. All patients had a durable remission from ITP after treatment (or spontaneously without treatment in two cases). Only one patient had another episode of ITP; her initial episode occurred 16 months after the last alemtuzumab dose, and then she experienced a second episode 7 years later that was responsive to treatment with corticosteroids.

Postmarketing surveillance has found the incidence of autoimmune cytopenias to be generally lower than in clinical trials for ITP (postmarketing estimated frequency: 0.58%), hemolytic anemia (0.05%), and pancytopenia (0.10%).^50^ Neutropenia in the postmarketing setting (0.48%) was higher than that of serious neutropenia in the clinical trials (0.2%), but lower than that of all neutropenia events in the clinical trials (1.7%).

In the clinical development program, four cases of autoimmune nephropathy occurred among 1486 patients exposed to alemtuzumab, giving a cumulative incidence of 0.27%. Overall, two cases were membranous glomerulonephritis, one case was glomerulonephritis with anti-glomerular basement membrane (GBM) antibodies, and one was anti-GBM disease. The patients with anti-GBM disease or with membranous glomerulonephritis with anti-GBM antibodies were treated with plasmapheresis, cyclophosphamide, and steroids. The patients with membranous glomerulonephritis were treated with angiotensin-converting enzyme inhibitors and diuretics. A total of three patients entered remission and recovered renal function, whereas the fourth had persistent proteinuria at the time of reporting; however, this patient’s membranous glomerulonephritis was resolved.^54^ In postmarketing surveillance, the estimated frequency of nephropathy is 0.13%.^50^

A single case report was published on a patient who developed type 1 diabetes 29 months after receiving the second course of alemtuzumab.^55^ The patient had a family history of type 1 diabetes and was prediabetic before initiating alemtuzumab. This case highlights the importance of a patient’s susceptibility to other autoimmune conditions as a component of risk assessment.

Importantly, in controlled studies, alemtuzumab efficacy and patient-reported QoL were unaffected by the occurrence of autoimmune AEs. Subgroup analyses performed on core study data showed that at Year 2, there were no significant differences in QoL improvements in alemtuzumab-treated patients with or without thyroid AEs,^56^ or with or without any autoimmune AE.^57^ In an analysis of 4-year data from the CARE-MS studies, ARR and the proportion of patients free of CDW were similar in subgroups of patients who had and had not experienced a thyroid AE.^58^

## Evolving understanding of alemtuzumab mechanism of action

Alemtuzumab selectively targets CD52, an antigen expressed highly on the surface of circulating T and B lymphocytes, cells that contribute directly to the underlying pathophysiology of MS. B-cells serve as highly specific antigen-presenting cells and provide costimulation to autoreactive T-cells, and antibodies contribute to demyelination and complement fixation.^59,60^ T-cells mediate damage to myelin and nerves.^61,62^ Regulatory T-cells (T_regs_) inhibit autoreactive T-cells through cell–cell interactions and the secretion of anti-inflammatory cytokines.^63,64^ In patients with MS, T_reg_ impairment enables activation of CD4^+^ T-cells including the T-helper (Th) 17 and Th1 cell subsets; in turn, Th17 and Th1 cells activate microglia to assume an inflammatory and neurotoxic phenotype.^65,66^

Alemtuzumab depletes circulating T and B lymphocytes through antibody-dependent, cell- and complement-mediated cytotoxicity,^67,68^ with the lowest cell counts observed at the first post-baseline assessment (1 month after treatment in CARE-MS I and II). Lymphocyte depletion is followed by a distinct pattern of T- and B-cell repopulation that begins within weeks, with B-cell counts returning to baseline levels within 6 months, whereas T-cell counts rise more slowly, generally approaching the lower limits of normal by 12 months.^10,69^ A recent multicenter follow up of 29 patients also found that the percentage of CD4+ T-cells (as a proportion of total lymphocytes) in the periphery did not return to baseline levels until 48 months later.^70^ There was no cumulative reduction in CD4+, CD8+, and CD19+ repopulation capacity with successive alemtuzumab courses.^71^

Recent results in an animal model suggested that alemtuzumab efficacy is related to the level of CD4^+^ T-cell depletion; however, the pattern of lymphocyte repopulation is key to the proposed alemtuzumab mechanism of action.^10,72^ Repopulation results in a relative increase of cells with memory and regulatory phenotypes and a decrease in cells with a proinflammatory signature.^73,74^ Th17 and Th1 cells remained low through at least 48 months following alemtuzumab treatment, with a concomitant decrease in proinflammatory cytokines associated with these cell types.^70^

Finally, although alemtuzumab has a minimal or transient effect on circulating monocytes, eosinophils, and basophils,^69^ recent data indicate that it may promote an immunoregulatory environment through an impact on other innate immune cells (e.g. dendritic cells) that play a role in MS. In patients with MS, dendritic cells are functionally abnormal, express upregulated levels of costimulatory molecules and proinflammatory cytokines, and stimulate proinflammatory cytokine secretion by effector T-cells.^65,75,76^ They may also interact with natural killer (NK) cells to induce selective CD56^bright^ NK cell expansion.^65,66,77^ The CD56^bright^ NK population exerts regulatory effects on autoreactive T-cells *via* cytotoxicity or suppression of T-cell proliferation.^78^ Alemtuzumab reduced the proportions of circulating dendritic cells (conventional and plasmacytoid subsets), and expanded the CD56^bright^ NK cell population, although the cytolytic function of NK cells was not altered.^41,79^ Together, the effects of alemtuzumab on T and B-cells, its effects on subsets of innate immune cells important in RRMS pathophysiology, and its overall rebalancing of the immune system may underlie its durable clinical effects (including disability improvement and slowing of BVL) and reduce the potential for relapse, ultimately delaying disease progression.

## Benefit–risk considerations for alemtuzumab use

A notable benefit of alemtuzumab therapy is improvement in preexisting disability, which may have a positive impact on long-term prognosis. Other important benefits are a reduction in the rate of BVL, achievement of annual NEDA, improvements in QoL, and a potentially low risk of progressing to SPMS.^4,5,21,32,38^ During the phase II and III core studies and continuing throughout the extension, these benefits were achieved in the absence of continuous treatment with regular, repeated dosing on a daily, every other day, thrice weekly, weekly, every other week, monthly, or every 6 month schedule. Furthermore, the majority of patients did not receive additional alemtuzumab or any other DMT after the initial 2-course regimen. This relative freedom from therapy, together with long-lasting clinical improvement or stability, comprehensive education for clinicians, patient education, and rigorous safety monitoring, may represent a shift in the standard by which therapeutic efficacy is judged.

Considerations of the potential benefits of alemtuzumab treatment must also be balanced by potential risks. The risks associated with alemtuzumab are well characterized and detectable with vigilant monitoring and, when identified early, are generally manageable. Follow up of up to 10 years confirmed that the risk for autoimmune AEs decreases substantially in the fourth year after the last alemtuzumab dose. This observation supports the rationale for the timeframe of the safety monitoring program (4 years after the last alemtuzumab dose, i.e. a minimum of 5 years in patients who require only two courses of alemtuzumab), which was effective in detecting autoimmune AEs during the clinical trials. In the case of thyroid autoimmune events, few were serious. Armed with a well characterized safety profile, a thorough education program, and careful monitoring procedures, clinicians are able to maximize benefits while minimizing treatment risks.

Alemtuzumab is a highly efficacious DMT that offers a new treatment principle for RRMS, but its pharmacodynamic effects are not reversible in the short term, and it may require both treatment courses before full clinical benefit is observed. The strong evidence from the clinical development program suggests it should be used relatively early in the disease course; due to its irreversible effects in the short term and potential side effects, benefit–risk ratio has to be evaluated individually in the context of other available treatments.

## Experience with switching to alemtuzumab from other DMTs

The most comprehensive available data set on outcomes of switching therapy to alemtuzumab comes from the CARE-MS patients who were randomized to SC IFNβ-1a in the core studies and, on entering the extension study, switched therapy to two courses of alemtuzumab.^80,81^ The protocol did not specify a washout period, but patients had ⩽2 months after discontinuing SC IFNβ-1a to receive alemtuzumab. Over 4 years of follow up after alemtuzumab initiation, ARR fell by approximately 70% compared with SC IFNβ-1a treatment, 81% (CARE-MS I) and 80% (CARE-MS II) of patients remained free of 6-month CDW, and 27% and 22% achieved 6-month CDI, respectively. The rate of BVL slowed after alemtuzumab initiation.^82^ No new safety signals arose in this cohort.

Data documenting outcomes in patients who switched to alemtuzumab from other DMTs are limited. An observational, multicenter study reported on 36 patients who initiated alemtuzumab after having inflammatory disease activity while on fingolimod therapy. Median fingolimod washout period was 6 weeks. Overall, nine of these patients experienced significant disease activity in the 12 months following alemtuzumab infusion.^83^ A total of eight patients were characterized by ⩾1 clinical relapse and radiologic disease activity, with the remaining patient by radiologic disease activity alone. All nine patients subsequently received the second course of alemtuzumab, after which eight patients were relapse-free (mean 6-month follow up). These data highlight the importance of administering the full treatment of two courses. The authors proposed that prolonged sequestration of autoreactive lymphocytes following fingolimod withdrawal shielded the cells from the biologic effect of alemtuzumab and that subsequent lymphocyte outflow elicited disease reactivation.^83^

In another observational study, patients (*n* = 16) who had responded to natalizumab, but discontinued because of the risk of PML, initiated alemtuzumab after a median washout of 70 days.^84^ Available brain MRI data at 6 and 12 months after alemtuzumab administration showed no signs of disease activity. Clinical evaluation at 6 and 12 months indicated stability, with no relapses or increase in EDSS. None of the patients experienced severe side effects or developed PML.

A case report was published in which two patients presented with apparent severe exacerbation of MS symptoms after alemtuzumab infusion; it was undetermined whether these were actual MS relapses or secondary autoimmunity directed toward the central nervous system. Both patients had ongoing disease activity despite prior treatments (most recently dimethyl fumarate and fingolimod) before switching to alemtuzumab. These cases were successfully managed with plasma exchange followed by rituximab administration.^85^ The authors proposed that these cases were B-cell-driven, given the effectiveness of plasma exchange plus rituximab, and that the timing of symptom onset coincided with B-cell repopulation post-alemtuzumab; however, whether these symptoms reflected MS exacerbation or a secondary autoimmunity was undetermined.

A second case report noted increased disease activity after switching to alemtuzumab in a patient with a treatment history of glatiramer acetate, fingolimod, natalizumab, and dimethyl fumarate (previous switches had been made due to disease progression or John Cunningham virus positivity).^86^ After 4 months from alemtuzumab initiation, the patient experienced unilateral dysesthesia and intermittent ataxia; brain MRI disclosed a tumefactive demyelinating lesion. The patient recovered spontaneously over 3 months.

## Real-world studies

Robust real-world studies are needed following marketing authorization to assess the short-and long-term effectiveness of DMTs in the population of patients with well characterized comorbidities and comedication who are eligible to receive treatment in clinical practice, and for whom patient monitoring is not constrained by protocol.^87,88^ Although more than 1400 patients have been treated with alemtuzumab in controlled clinical trials, data on the use of alemtuzumab and outcomes of treatment under clinical practice conditions will provide valuable additional information on the real-world efficacy and safety of this agent. Real-world data to date are limited, given that marketing approval was granted only 3 years ago in Europe and 2 years ago in the US.

A recent, observational study of the MSBase cohort provided insights into comparative effectiveness of alemtuzumab and other DMTs for patients with RRMS. Of 15,783 patients with MS, 4332 received either alemtuzumab (*n* = 189), IFNβ (*n* = 2155), fingolimod (*n* = 828), or natalizumab (*n* = 1160) between 1994 and 2016.^89^ Patients had a mean disease duration of roughly 3 years, with an EDSS score of 3.0 and approximately two relapses in the 12 months before baseline. In an analysis of propensity score-matched patients, ARR with alemtuzumab was lower than with IFNβ [0.19 (95% CI, 0.14–0.23) *versus* 0.53 (0.46–0.61), *p* < 0.0001] or fingolimod [0.15 (0.10–0.20) *versus* 0.34 (0.26–0.41), *p* < 0.0001], and similar to ARR with natalizumab [0.20 (0.14–0.26) *versus* 0.19 (0.15–0.23), *p* = 0.78]. Probabilities of disability accumulation were similar among all four therapies.^89^

In a chart review study in the UK, 100 patients with aggressive RRMS and a poor prognosis were treated with alemtuzumab since 2000 and followed up for a mean of 6.1 years.^90^ Alemtuzumab use was off-label prior to its approval for RRMS and, until 2006, the dosage given was higher (24–30 mg/day) than the currently approved dosage (12 mg/day). ARR decreased from 2.1 pretreatment to 0.2 post-treatment; a low ARR was maintained up to 8 years. Mean EDSS change from the pretreatment level was +0.14, 27% of patients experienced CDW, and 25% had CDI. The alemtuzumab safety profile in this cohort was consistent with the clinical trial experience, with the exceptions of five cases of cervical dysplasia and three cases of monoclonal gammopathy of uncertain significance.

TREAT-MS is an ongoing, open-label, uncontrolled, multicenter, prospective, noninterventional study documenting the alemtuzumab experience in approximately 3200 RRMS patients from 400 sites in Germany.^91^ In this study, the MS software-based documentation system, MSDS3D, is used to guide physicians through the complex processes of diagnosis and treatment.^92,93^ Patients complete questionnaires to evaluate satisfaction with treatment, cognition, state of health, health-related QoL, and ability to work. In addition, physicians provide an assessment of patients’ state of health and disease-related QoL. Safety data are also being collected. Preliminary analyses of baseline characteristics of the enrolled population showed that TREAT-MS patients have more severe MS disease than patients in the phase II and III studies, with a longer average disease duration, higher EDSS score, and more relapse activity. Thus, this cohort will provide new insights on alemtuzumab efficacy in more severe MS.

In a real-world, ongoing, observational cohort of 250 patients who received alemtuzumab at a single MS treatment center in the US, interim analysis showed that during 4–12-month follow up, there was a marked absence of clinical worsening.^94^ IARs were common but generally mild, and no cases of PML were reported. The majority (*n* = 200) of patients in this cohort had discontinued natalizumab before initiating alemtuzumab. It is possible that alemtuzumab helped attenuate disease re-emergence after natalizumab discontinuation, although the lack of a control arm precludes this conclusion. During the 6 months following natalizumab discontinuation and alemtuzumab initiation, none of the patients relapsed; EDSS score improved in 69/162 (43%) patients and worsened in 2/162 (1%).^94^

## Outlook

In addition to the aforementioned completed and ongoing real-world studies, longer-term data on outcomes in patients from the clinical development program are being collected. Patients completing the CARE-MS extension study are being followed up for an additional 5 years in the TOPAZ study [ClinicalTrials.gov identifier: NCT02255656].^95^ At the conclusion of TOPAZ, the CARE-MS cohort will have been followed up for a total of 11 years, and patients from CAMMS223 for 15 years.

TOPAZ is accompanied by many other phase IV studies currently being conducted across Europe and the Americas, focusing on areas including infusion management (EMERALD), QoL and other patient-reported outcomes (LEMQoL, PRO-ACT, LemVida, REALITY), safety (PASS), and cognition (LemCOG). In addition, an exploratory, independent phase IV study has been initiated to identify markers for treatment response and autoimmune AE risk stratification. This analysis of immunologic parameters will help to improve patient selection and elucidate the mechanism of action of alemtuzumab.^96^ Thus the knowledge base about alemtuzumab treatment of RRMS will continue to grow as data emerge on longer-term efficacy and safety (>6 years), mechanism of action, patient perspectives, and other nontraditional endpoints.
